# An increase of interleukin-33 serum levels after coronary stent implantation is associated with coronary in-stent restenosis

**DOI:** 10.1016/j.cyto.2014.02.014

**Published:** 2014-06

**Authors:** Svitlana Demyanets, Ioannis Tentzeris, Rudolf Jarai, Katharina M. Katsaros, Serdar Farhan, Anna Wonnerth, Thomas W. Weiss, Johann Wojta, Walter S. Speidl, Kurt Huber

**Affiliations:** a Department of Internal Medicine II, Medical University of Vienna, Währinger-Gürtel 18-20, 1090 Vienna, Austria; b Department of Laboratory Medicine, Medical University of Vienna, Währinger-Gürtel 18-20, 1090 Vienna, Austria; c 3rd Medical Department for Cardiology and Emergency Medicine, Wilhelminenhospital, Montleartstraße 37, 1160 Vienna, Austria; d Ludwig Boltzmann Cluster for Cardiovascular Research, Währinger-Gürtel 18-20, 1090 Vienna, Austria

**Keywords:** Interleukin-33, Restenosis, Coronary artery disease, Myocardial infarction, Percutaneous coronary intervention

## Abstract

The study aim was to determine the predictive value of interleukin (IL)-33, a recently described member of the IL-1 family of cytokines, for the development of in-stent restenosis (ISR). IL-33 serum levels were measured in 387 consecutive patients undergoing percutaneous coronary intervention (PCI) of whom 193 had stable angina, 93 non-ST elevation myocardial infarction (NSTEMI), and 101 ST-elevation MI (STEMI), respectively. Blood was taken directly before and 24 h after stent implantation. The presence of ISR was initially evaluated by clinical means after six to eight months. When presence of myocardial ischemia was suspected, coronary angiography was performed to confirm the suspected diagnosis of ISR. Clinical ISR was present in total in 34 patients (8.8%). IL-33 was detectable in 185 patients and was below detection limit in 202 patients. In patients with decreased IL-33 (*n* = 95), unchanged or non-detectable levels (*n* = 210) or increased levels of IL-33 after PCI (*n* = 82), ISR-rate was 2.1%, 9.5% and 14.6%, respectively (*p* < 0.05). Accordingly, patients with ISR showed a significant increase of IL-33 upon PCI (*p* < 0.05). This association was independent from clinical presentation and risk factors as well as numbers and type of stents. In patients with both stable and unstable coronary artery disease, an increase of IL-33 serum levels after stent implantation is associated with a higher rate of in-stent restenosis.

## Highlights

• An association between IL-33 and restenosis in coronary artery disease exists.
• IL-33 increase after stent implantation is associated with a higher rate of restenosis.
• IL-33 estimation before and after PCI could determine patients at risk.

## 1 Introduction

Restenosis after percutaneous coronary intervention (PCI) remains an unsolved clinical problem and certain patients appear to be at increased risk of developing restenotic complications. Defining the subgroups of patients at increased or decreased risk for in-stent restenosis (ISR) would be of massive utility for patient risk stratification and for the understanding of underlying molecular and cellular mechanisms [1,2].

Restenosis is a multifaceted disease and pathophysiological mechanisms involved are thought to comprise inflammation, proliferation and extracellular matrix remodeling. Inflammation seems to play a central role in the pathogenesis of ISR [1,3,4]. Both “classical” inflammation, mediated by neutrophils, monocytes and T helper type 1 (Th1) lymphocytes, and “allergic” inflammation, mainly mediated by eosinophils and T helper type 2 (Th2) lymphocytes, are implicated in restenotic reactions [2].

Different inflammatory markers for restenosis have been identified and include complement components C5a and C3a [5], tumor necrosis factor (TNF)-α [6] and interleukin (IL)-3 [7]. Circulating levels of matrix metalloproteinase (MMP)-2, MMP-9, plasminogen activator inhibitor (PAI)-1 and soluble Fas and Fas ligand were also shown as a predictive marker for ISR by our group and others [8–12].

IL-33 is the most recently described member of the IL-1 family of cytokines and is a ligand for the ST2 receptor [13]. IL-33 is expressed intracellularly predominantly by stromal cells such as endothelial and epithelial cells as well as smooth muscle cells and fibroblasts [14–16]. IL-33 is believed to be released during necrosis but kept intracellular during apoptosis where it is inactivated by caspases [17,18]. Therefore, IL-33 is recognized as a dual function cytokine that acts either intracellular to regulate gene transcription or extracellular via binding to ST2. Upon release, IL-33 was shown to be recognized by different immune and non-immune ST2-expressing cells. In such a way IL-33 integrates both innate and adaptive immunity in a unique fashion via activation of eosinophils, basophils, mast cells, innate lymphoid cells, and Th2 lymphocytes [16,19]. Thus, IL-33 might function as an alarmin, a danger signal belonging to the larger family of damage-associated molecular pattern (DAMP) molecules [15,20].

IL-33 is involved in the pathogenesis of different inflammatory and allergic disorders such as rheumatoid arthritis, asthma, psoriasis and ulcerative colitis [21–23]. A precise role of IL-33 in the pathogenesis of cardiovascular diseases is still not well defined. Dhillon et al. measured IL-33 levels in patients with myocardial infarction and found that elevated IL-33 was associated with increased mortality in ST-elevation myocardial infarction (STEMI) [24], but was not related to adverse events in non–ST-elevation myocardial infarction (NSTEMI) patients [25].

We propose that IL-33 may be an important player in the pathogenesis of ISR after stent implantation and therefore circulating levels of IL-33 could serve as a biomarker for the development of ISR. The aim of this study was to test whether an increase of IL-33 after PCI is associated with an increased rate of ISR.

## 2 Methods

### 2.1 Study population

Blood samples were taken from 387 consecutive patients undergoing PCI. From these patients 193 had stable angina, 93 NSTEMI, and 101 STEMI, respectively. The PCIs were performed according to standard techniques by experienced interventionalists only. Exclusion criteria were presence of autoimmune diseases, chronic infections, hepatic or renal disorders. Aspirin and unfractionated heparin were administered per standard practice. Clopidogrel therapy was started either on the day before angiography or immediately after stent implantation with 300 mg. After the procedure, patients were maintained on aspirin 100 mg indefinitely, and clopidogrel 75 mg according to guidelines of European Society of Cardiology (ESC). Other medications such as beta-blockers and angiotensin-converting-enzyme inhibitors were given as appropriate. Statin therapy was routinely administered to all patients according to international guidelines. At inclusion time new antiplatelet drugs like ticagrelor and prasugrel were not yet available. After enrollment, patients remained in the hospital for at least 48 h. Informed consent was obtained from each patient. The study protocol conforms to the ethical guidelines of the 1975 Declaration of Helsinki as reflected in a priori approval by the ethics committee of the city of Vienna, Austria.

### 2.2 Blood samples

Two blood samples were taken directly before PCI (at baseline) and 24 h after PCI. Blood drawing was performed under fasting conditions whenever possible (in stable patients and 24 h after the event in patients with acute events). Venous blood was drawn from the antecubital vein with minimal tourniquet pressure into serum separator tubes. Samples were allowed to clot for 30 min (min) before centrifugation (4 °C; 3000 g for 15 min) and stored at −80 °C until use.

### 2.3 Laboratory measurements

IL-33 was measured with a specific enzyme-linked immunosorbent assay (ELISA; R&D Systems; Minneapolis, MN, USA). The minimum detection limit of the assay was 23.4 pg/mL. The sensitivity of the assay is expressed as minimum detectable dose (MDD) and mean MDD was 0.5 pg/mL. For calculation of the intra-assay coefficient of variability (CV) three samples of known concentration were tested twenty times on one plate and mean ± SD was assessed as 3.2 ± 1.0%. For calculation of the inter-assay CV three samples of known concentration were tested in twenty separate assays and mean ± SD was assessed as 5.3 ± 0.5%. Laboratory determinations were performed by investigators that were blinded to clinical characteristics and patients’ outcome.

### 2.4 Angiographic definitions

Maximal lumen stenosis was measured within the stent and within the 5-mm proximal and distal edges of the stent. All measurements were performed by the same investigator that was blinded to all laboratory results.

### 2.5 End points

All patients were reevaluated clinically for recurrent anginal symptoms after six to eight months. Patients with clinical signs of restenosis underwent re-angiography. The primary end point of the study was the need for target lesion revascularization due to restenosis in the presence of symptoms or objective signs of ischemia during the follow-up.

### 2.6 Statistical analysis

Sample size calculation was based on the hypothesis that IL-33 increase is associated with at least a 15% higher restenosis rate as compared to patients with IL-33 decrease. Sample size calculation revealed that we would need at least 75 patients per group to detect a difference with a power of 80% and significance level (two-tailed) of 0.05 [26]. As IL-33 was not detectable in approximately 50% of patients we increased the sample size accordingly. Continuous variables are expressed as mean ± SD or as median, interquartile range. Categorical variables are summarized as counts and percentages and were compared by the chi-square or by Fisher exact test. Continuous variables were compared using Student’s *t*-test when normally distributed and by Mann–Whitney-*U* test when not normally distributed. Spearman correlation was used to determine the correlation between level of IL-33 and cardiovascular risk factors. Multivariate analysis was performed with the logistic regression model in which restenosis was used as dependent variable and potentially confounding baseline variables were used as independent variables. Baseline variables were selected for the model if they (a) had either a clinically plausible relation with the outcome or (b) appeared to be imbalanced between patients with and without restenosis indicated by a *p*-value < 0.20. A value of *p* < 0.05 (two-tailed) was considered statistically significant. All statistical analyses were performed with the statistical software package SPSS version 18.0 (SPSS, Inc., Chicago, Illinois).

## 3 Results

### 3.1 Patient characteristics

BMS were used in 283 and DES were used in 104 patients. Clinical ISR was present in total in 34 patients (8.8%; 7 DES and 27 BMS). Target lesion revascularization was performed in all 34 patients. Baseline demographic data are shown in Table 1. Patients with and without restenosis at follow-up showed no significant differences in baseline clinical characteristics and cardiovascular risk factors. However, patients in the restenosis group tended to have a higher prevalence of hypertension, family history of CAD, hyperlipidaemia and peripheral artery occlusive disease (PAOD) (Table 1). There were no significant differences in baseline angiographic characteristics (Table 2).

### 3.2 IL-33 levels and cardiovascular risk factors

IL-33 was detectable in 185 patients (ranged from 0.4 to 2180.0 pg/mL) and was below detection limit in 202 patients. Median IL-33 levels before (*p* = 0.40) or after (*p* = 0.60) PCI were not different in patients with stable CAD, NSTEMI or STEMI. IL-33 levels at baseline were associated with age (*r* = 0.11, *p* < 0.05) and correlated inversely with estimated glomerular filtration rate (eGFR, *r* = −0.10, *p* < 0.05). Baseline IL-33 was significantly higher in females (*p* < 0.05) and non-smokers (*p* < 0.005). Interestingly, IL-33 levels before PCI statistically significantly correlated (*p* < 0.05) with percent stenosis of the culprit lesion. However, this correlation was only weak (*r* = 0.123).

### 3.3 Decreased IL-33 serum levels after coronary stent implantation are associated with lower rate of ISR

**Fig. 1:**
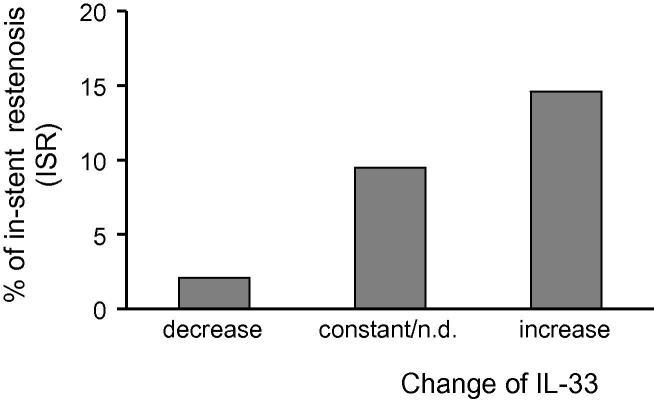
Restenosis rate according to the change of IL-33 serum levels. IL-33 serum levels were measured in the patients before and 24 h after PCI by ELISA as described under “Section 2”. In patients with decreased IL-33, unchanged or non-detectable (n.d.) levels or increased levels of IL-33 after PCI, in-stent restenosis (ISR) rate was 2.1%, 9.5% and 14.6%, respectively.

In patients with decreased IL-33 (*n* = 95), unchanged or non-detectable (n.d.) levels (*n* = 210) or increased levels of IL-33 after PCI (*n* = 82), the respective ISR-rate was 2.1%, 9.5% and 14.6% (*p* < 0.05) (Fig. 1). IL-33 serum levels before or after PCI were not associated with ISR at follow-up (*p* = 0.901 and *p* = 0.790, respectively).

### 3.4 Change in IL-33 serum levels in patients with and without restenosis

**Fig. 2:**
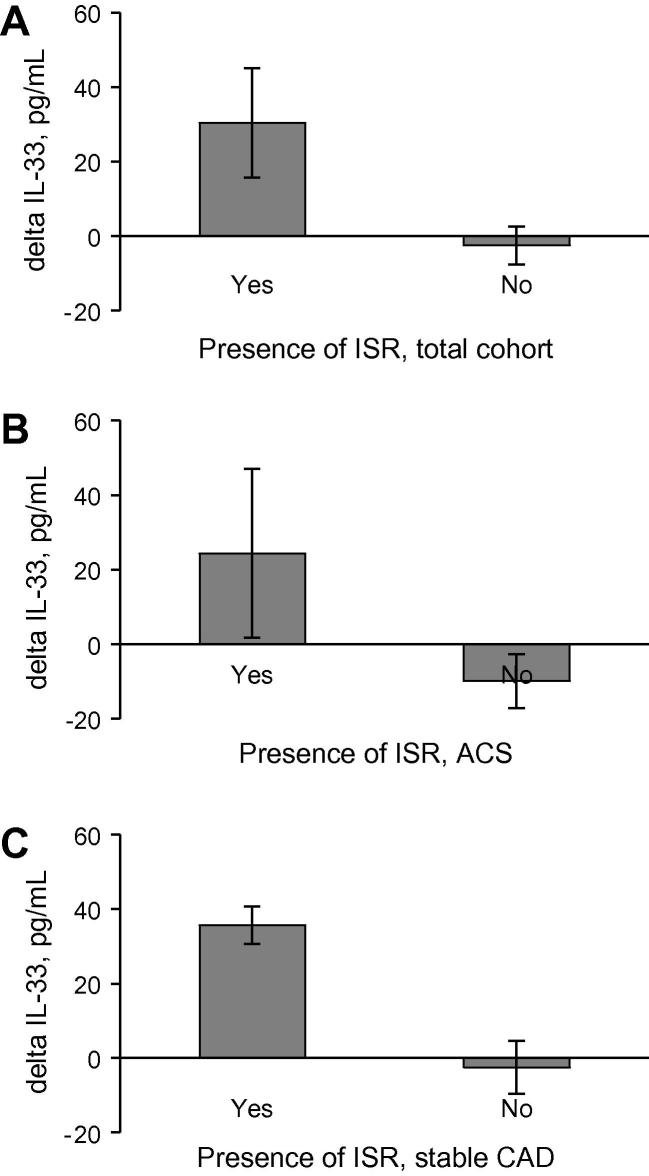
Change in IL-33 levels according to the presence of ISR. IL-33 serum levels were measured in the patients before and 24 h after PCI by ELISA as described under “Section 2”. Changes in IL-33 serum levels are shown in accordance with presence (Yes) or absence (No) of in-stent restenosis (ISR) in the entire cohort (panel A) or patients with acute coronary syndrome (ACS; panel B) or stable coronary artery disease (CAD, panel C).

Accordingly, patients with ISR showed a significant increase of IL-33 upon PCI (*p* < 0.05) in the entire cohort (Fig. 2A) as well as in the patients with acute coronary syndrome (ACS; Fig. 2B) or stable CAD (Fig. 2C). This association was independent of clinical presentation and risk factors as well as numbers and type of stents as assessed by a multivariate regression model (Table 3).

## 4 Discussion

In the present study we found that a decrease of IL-33 serum levels after stent implantation is associated with a lower rate of in-stent restenosis after PCI in patients with both stable and unstable CAD. Consequently, patients with ISR showed a significant increase of IL-33 upon PCI. Interestingly, levels of IL-33 before PCI correlated positively with grade of stenosis in target vessel at baseline, although this correlation was only weak. Multiple logistic regression demonstrated that changes in IL-33 levels independently predicted the occurrence of restenosis after PCI.

Our report is the first that showed the predictive value of changes in IL-33 serum levels for in-stent restenosis after vascular intervention. The activation status of the inflammatory system has been suggested to play an important role in predicting restenosis [1,2,4]. Previously, different inflammatory markers for restenosis have been identified by our group and others including complement components C3a and C5a [5] and cytokines TNF-α [6], IL-3 [7], IL-6 [27], and IL-10 [28]. Moreover, ISR after stent implantation was shown to be related to the levels of circulating MMP-2, MMP-9, PAI-1 and soluble Fas and Fas ligand [8–12].

IL-33 is a recently described member of the IL-1 family of cytokines, which also includes IL-1α, IL-1β and IL-18 [13]. IL-33 is predominantly expressed by stromal cells such as endothelial, epithelial and smooth muscle cells [14,21]. Cell damage can induce necrosis and release of IL-33 [21]. IL-33 was shown to be released by endothelial cells after damage or injury [17]. We showed recently that nuclear IL-33 is released from necrotic human coronary artery smooth muscle cells, human adult cardiac myocytes and cardiac fibroblasts in vitro [14].

Upon release, IL-33 was shown to be recognized by different immune cells such as Th2 cells, mast cells, basophils, eosinophils, and to activate these cells [22]. Generally, IL-33 is predominantly associated with the Th2-related immune response [13]. However, IL-33 is also able to enhance the production of the Th1 cytokine interferon-γ (IFN-γ) by natural killer cells and invariant natural killer T cells [29]. ST2L, the transmembrane receptor for IL-33, is also expressed on macrophages, and IL-33 was shown to enhance lipopolysaccharide-induced TNF-α, IL-6 and IL-1β production from macrophages [30]. Moreover, IL-33 enhances adhesion and survival of mast cells, eosinophils and basophils, as well as the release of different cytokines from these cells [21,22]. In this respect, it is of interest that eosinophilic infiltration was found in case of in-stent restenosis, and eosinophils have been identified as important modulators of restenosis after stent implantation [31,32]. Recent findings have also implicated mast cells in the pathogenesis of cardiovascular disorders [33]. Therefore, it is possible to hypothesize that IL-33, released by stromal cells such as endothelial and smooth muscle cells during damage or injury, via activation of different immune cells might play an important role in the process of restenosis. It should be emphasized, that IL-33 is considered to be a so called DAMP or alarmin, which guides the immune response after cellular injury [20].

The paradigm of coronary stenting, leading to vascular injury and resulting in an inflammatory response, and subsequent in-stent restenosis shares many similar features of the inflammatory cascade that propagates atherosclerosis [34]. Experimental studies suggest a possible implication of IL-33 in development and progression of atherosclerosis. IL-33 and its receptor ST2 are expressed in murine and human atherosclerotic lesions [35,36]. Although injection of IL-33 into apolipoprotein E (ApoE) knockout mice with ongoing atherosclerosis reduced atherosclerotic lesions in thoracic aorta [35], treatment of human endothelial cells with IL-33 increased the expression of adhesion molecules and production of the chemokine monocyte chemoattractant protein (MCP)-1 and promoted adhesion of leukocytes to endothelial cells [36], all of which are features of endothelial activation and are recognized as an early step in the development of atherosclerosis [34]. Activation of leukocytes is seen as an essential step also in the development of restenosis after PCI [4].

The association between IL-33 and ISR might be explained by the direct proinflammatory effects of IL-33 on endothelial cells. Additional to the upregulation of intercellular adhesion molecule-1, vascular cell adhesion molecule-1, and endothelial selectin, IL-33 also induces IL-6, IL-8, and MCP-1 in endothelial cells [36,37]. Furthermore, IL-33 was shown to induce the activation of nuclear factor-*κ*B (NF-*κ*B), a transcription factor regulating cytokine synthesis and inflammatory activation, in human endothelial cells, mast cells, eosinophils, and basophils [16,36].

Angiogenesis is recognized as an important factor in the development and progression of restenosis. Restenotic lesions develop intimal neovascularization, apparently necessary for neointimal growth [38]. Recently, it was also demonstrated that IL-33 induces vascular permeability and angiogenesis in human endothelial cells [39].

There are no data available on possible effects of IL-33 on smooth muscle cells migration and proliferation. However, as human coronary artery smooth muscle cells express only minor amounts of the receptor for IL-33, ST2L, as compared to endothelial cells [14,35], it could be hypothesized that IL-33 has only minor direct effects on the biology of smooth muscle cells, and impacts on the pathophysiology of the vasculature mostly via its effects on endothelial cells and immune hematopoietic cells.

Binding of extracellular IL-33 to the heterodimeric receptor complex consisting of transmembrane ST2L and IL-1 receptor accessory protein induces recruitment of Myd88 and activation of NF-*κ*B and mitogen-activated protein kinases [13,21]. Interestingly, a recent study showed that inhibition of MyD88 or IL-1 receptor signalling reduces neointima formation in response to vascular injury in mice [40].

Although increased levels of the soluble receptor for IL-33, sST2, is a marker of poor prognosis in patients with heart failure and myocardial infarction [21], the prognostic value of circulating IL-33 in cardiovascular disease was not studied extensively. Recently, Dhillon et al. measured IL-33 levels in 577 patients with NSTEMI and found no relation of IL-33 levels to adverse events such as all-cause mortality, heart failure hospitalization, and reinfarction [25]. However, the same group found that in STEMI patients elevated IL-33 was associated with increased 30-days and 1-year mortality [24]. Moreover, circulating IL-33 levels were shown to be increased in patients with different immune-inflammatory diseases such as rheumatoid disorders [23,41], systemic sclerosis [42], and ulcerative colitis [43].

Some limitations of the present study have to be acknowledged. As the primary endpoint of this study was need for target lesion revascularization due to restenosis in the presence of symptoms or objective signs of ischemia during the follow-up we can not exclude that routine follow-up angiography would have revealed additional cases of ISR. In addition, despite inclusion of 387 patients only 34 patients (8.8%) developed clinical signs of ISR. Therefore, larger studies are needed to confirm our results in additional patient cohorts before any clinical conclusions can be drawn. In addition, we included patients with DES and BMS. However, multivariate analysis revealed that the association between IL-33 and the development of ISR is independent of stent type. Further, our study is necessarily of an observational nature. Accordingly, our results may be explained by unmeasured confounding factors. Therefore, we tried to control for baseline imbalances by multivariate modeling. However, the possibility of residual or undetected confounding is small but cannot be ruled out completely. In addition, IL-33 was not detectable in 202 patients before or after the intervention. Similar to our findings, Dhillon et al. found that in more than half of the patients IL-33 levels were below the lower detection limit of the assay used [24,25]. Interestingly, the patients with IL-33 below the detection limit of the assay, showed an intermediate ISR rate, suggesting that these low levels of IL-33, have no or only little impact on the development of ISR.

## 5 Conclusions

In conclusion, we provide evidence that IL-33 might be involved in the pathogenesis of yet another inflammatory pathology, namely in-stent restenosis after coronary stent implantation [1]. A recent review emphasized the growing importance of inflammatory biomarkers that allow identification of individuals at high risk to develop ISR after coronary intervention and stent implantation for the management of these patients [2]. Based on our findings that an increase in IL-33 levels after coronary stent placement is associated with ISR, we propose here IL-33 as a new circulating biomarker and a tool to identify patients with an increased risk of developing in-stent restenosis.

## Figures and Tables

**Table 1:** Baseline characteristics of study population.

	Total (*n* = 387)	Restenosis (*n* = 34)	No restenosis (*n* = 353)	*p*-Value
Age (yrs)	64.6 ± 12.7	62.8 ± 10.6	64.8 ± 12.9	0.39
Male sex, *N* (%)	257 (66.4%)	23 (67.6%)	234 (66.3%)	1.00
Hypertension, *N* (%)	288 (74.4%)	27 (79.4%)	261 (73.9%)	0.55
Family history of CAD, *N* (%)	31 (8.0%)	4 (11.8%)	27 (7.6%)	0.34
Smoker, **N** (%)	113 (29.2%)	9 (26.5%)	104 (29.5%)	0.35
Hyperlipidaemia, **N** (%)	296 (76.5%)	28 (82.4%)	268 (75.9%)	0.53
BMI, (kg m^−2^)	27.7 ± 4.4	27.2 ± 3.9	27.8 ± 4.5	0.49
Diabetes, **N** (%)	82 (21.2%)	7 (20.6%)	75 (21.2%)	1.00
PAOD, **N** (%)	19 (4.9%)	3 (8.8%)	16 (4.5%)	0.23
eGFR (mL/min)	89.9 ± 62.8	88.2 ± 37.3	89.9 ± 65.8	0.88

Characteristics of the total cohort (*n* = 387) and patients with restenosis (*n* = 34) and without restenosis (*n* = 353) according to age, sex, body mass index (BMI), estimated glomerular filtration rate (eGFR), presence of hypertension, family history of coronary artery disease (CAD), hyperlipidaemia, diabetes, peripheral arterial occlusive disease (PAOD), and smoking status.

**Table 2:** Angiographic and interventional characteristics of study population.

	Total (*n* = 387)	Restenosis (*n* = 34)	No restenosis (*n* = 353)	*p*-Value
*Clinical presentation, N (%)*				0.22
Stable CAD	193 (49.9%)	18 (52.9%)	175 (49.6%)	
NSTEMI	93 (24.0%)	11 (32.4%)	82 (23.2%)	
STEMI	101 (26.1%)	5 (14.7%)	96 (27.2%)	

*Target vessel, N (%)*				0.45
LAD	159 (41.2%)	13 (38.2%)	146 (41.5%)	
LCx	91 (23.6%)	8 (23.5%)	83 (23.6%)	
RCA	129 (33.4%)	11 (32.4%)	118 (33.5%)	
Vein graft	7 (1.8%)	2 (5.9%)	5 (1.4%)	

*Vessel disease, N (%)*				0.36
1-VD	202 (52.2%)	18 (52.9%)	184 (52.1%)	
2-VD	108 (27.9%)	7 (20.5%)	101 (28.6%)	
3-VD	77 (19.9%)	9 (26.5%)	68 (19.2%)	
*Number of stents*	1.4 ± 0.6	1.4 ± 0.7	1.4 ± 0.6	0.51
*Percent stenosis*	86.4 ± 10.7	84.6 ± 13.7	86.6 ± 10.4	0.41

*Type of stent, N (%)*				0.26
BMS	283 (73.1%)	27 (79.4%)	256 (72.5%)	
DES	104 (26.9%)	7 (20.6%)	97 (27.5%)	

Characteristics of the total cohort (*n* = 387) and patients with restenosis (*n* = 34) and without restenosis (*n* = 353) according to clinical presentation, target vessel, number of diseased vessel, number and type of stents, and percent of stenosis. CAD denotes coronary artery disease, NSTEMI non-ST-elevation myocardial infarction, STEMI ST-elevation myocardial infarction, LAD left anterior descending artery, LCx left circumflex artery, RCA right coronary artery, VD vessel disease, BMS bare metal stent, DES drug eluting stent.

**Table 3:** Logistic regression model assessing the risk of restenosis after stent implantation.

	Odds ratio	95% Confidence interval	*p*-Value
*Univariate*			
IL-33 decrease	1.0	–	–
IL-33 constant, n.d.	4.90	1.12–21.39	0.035
IL-33 increase	7.97	1.73–36.77	0.008

*Adjusted for clinical characteristics* (*age, diabetes, smoking, hypertension*)			
IL-33 decrease	1.0	–	–
IL-33 constant, n.d.	4.91	1.12–21.49	0.035
IL-33 increase	8.19	1.77–37.93	0.007

*Adjusted for angiographic and interventional characteristics* (*presence of ACS, number of stents, type of stents, percent stenosis*)			
IL-33 decrease	1.0	–	–
IL-33 constant, n.d.	4.69	1.06–20.67	0.041
IL-33 increase	7.78	1.65–36.57	0.009

Multivariate analysis was performed with the logistic regression model in which restenosis was used as dependent variable and clinical characteristics (age, diabetes, smoking, hypertension) or angiographic and interventional characteristics (presence of acute coronary syndrome (ACS), number of stents, type of stents, percent stenosis) were used as independent variables. n.d. denotes not detectable.
